# Diagnostic accuracy of quantitative flow ratio (QFR) and vessel fractional flow reserve (vFFR) estimated retrospectively by conventional radiation saving X-ray angiography

**DOI:** 10.1007/s10554-020-02133-8

**Published:** 2021-01-16

**Authors:** Chongying Jin, Anantharaman Ramasamy, Hannah Safi, Yakup Kilic, Vincenzo Tufaro, Retesh Bajaj, Guosheng Fu, Anthony Mathur, Christos V. Bourantas, Andreas Baumbach

**Affiliations:** 1 Department of Cardiology, Sir Run Run Shaw Hospital, School of Medicine, Zhejiang University, Zhejiang, China; 2 Department of Cardiology, Barts Heart Centre, Barts Health NHS Trust, London, UK; 3 William Harvey Research Institute, Barts Heart Centre, Queen Mary University of London, West Smithfield, London, EC1A 7BE UK; 4 Institute of Cardiovascular Sciences, University College London, London, UK

**Keywords:** QFR, vFFR, Fractional flow reserve, Angiography

## Abstract

**Background:**

Angiography derived FFR reveals good performance in assessing intermediate coronary stenosis. However, its performance under contemporary low X-ray frame and pulse rate settings is unknown. We aim to validate the feasibility and performance of quantitative flow ratio (QFR) and vessel fractional flow reserve (vFFR) under such angiograms.

**Methods:**

This was an observational, retrospective, single center cohort study. 134 vessels in 102 patients, with angiograms acquired under 7.5fps and 7pps mode, were enrolled. QFR (fQFR and cQFR) and vFFR were validated with FFR as the gold standard. A conventional manual and a newly developed algorithmic exclusion method (M and A group) were both evaluated for identification of poor-quality angiograms.

**Results:**

Good agreement between QFR/vFFR and FFR were observed in both M and A group, except for vFFR in the M group. The correlation coefficients between fQFR/cQFR/vFFR and FFR were 0.6242, 0.5888, 0.4089 in the M group, with r_vFFR_ significantly lower than r_fQFR_ (p = 0.0303), and 0.7055, 0.6793, 0.5664 in the A group, respectively. AUCs of detecting lesions with FFR ≤ 0.80 were 0.852 (95% CI 0.722–0.913), 0.858 (95% CI 0.778–0.917), 0.682 (95% CI 0.586–0.768), for fQFR/cQFR/vFFR in the M group, while vFFR performed poorer than fQFR (p = 0.0063) and cQFR (p = 0.0054). AUCs were 0.898 (95% CI 0.811–0.945), 0.892 (95% CI 0.803–0.949), 0.843 (95% CI 0.746–0.914) for fQFR/cQFR/vFFR in the A group. AUC_vFFR_ was significantly higher in the A group than that in the M group (p = 0.0399).

**Conclusions:**

QFR/vFFR assessment is feasible under 7.5fps and 7pps angiography, where cQFR showed no advantage compared to fQFR. Our newly developed algorithmic exclusion method could be a better method of selecting angiograms with adequate quality for angiography derived FFR assessment.

**Supplementary Information:**

The online version contains supplementary material available at 10.1007/s10554-020-02133-8.

## Introduction

Fractional flow reserve (FFR) is regarded as the gold standard for the assessment of intermediate coronary artery stenoses (diameter stenosis (DS) 30–90%). An FFR ≤ 0.80 is regarded to represent functionally significant stenosis where revascularization is recommended. However, invasive pressure wire assessment requires a wire to be advanced through the lesion to the distal vessel. This comes with procedural risks along with increased time and cost. Adenosine is the most commonly used hyperaemic agent for invasive FFR assessment which itself is associated with the small risk of dyspnea, arrhythmia and cost. Therefore, although most guidelines have recommended FFR evaluation of intermediate coronary stenosis lesions to guide revascularization, only 15% of these lesions are evaluated by FFR in daily clinical practice [1–4].

Over the last few years, several angiography-derived virtual FFR assessment technologies have been developed. These are based on vessel geometry derived from 3 dimensional-quantitative coronary angiographies (3D-QCA), processed either by computational fluid dynamics (CFD) methods or using a mathematical formula which calculates the pressure drop across the lesion [5]. At present, three of these technologies are commercially available (Quantitative Flow Ratio (QFR), Medis medical imaging system bv., Netherland; FFRangio, Cathworks Ltd, Israel and vessel fractional flow reserve (vFFR), Pie Medical Imaging, Netherland) which have been validated against invasive FFR. All three technologies have shown a strong correlation with FFR measurements and good accuracy in predicting functionally significant coronary stenosis [6–12].

As angiography-derived computational FFR is based on 3D-QCA reconstruction of the interrogated vessel, good quality angiographic projections are required for accurate assessment of functional coronary stenosis. Over the last decade, several radiation reduction techniques have been implemented to reduce radiation exposure in the cardiac catheterization suite. This can either be done via reducing the X-ray frame rate, pulse or a combination of both [13, 14]. However, radiation reduction comes at a cost on angiographic image quality with a decreased signal to noise ratio. [15]. The acquired angiographic images may provide sufficient diagnostic information to guide percutaneous coronary intervention (PCI) but whether angiographic derived FFR based on these images allow accurate estimation of functional stenosis is unknown. This is important for the retrospective analysis of angiograms, which have been obtained in clinical practice with contemporary radiation reduction parameters.

In this study, we focused on the feasibility and diagnostic performance of two commercially available technologies, QFR and vFFR against invasive FFR when the coronary angiography has been performed using radiation-save mode (7.5 frames per second (fps) and 7 pulses per second (pps)), and try to develop a reasonable algorithm to select angiograms with adequate image quality under such mode for further angiography-derived computational FFR analysis.

## Methods

### Study design and patient population

The present study is a single centre, observational, retrospective study in which the diagnostic performance of both QFR and vFFR were evaluated and compared with invasive FFR. Patients (age > 18 years old) with at least one native coronary artery with intermediate stenosis (diameter stenosis (DS) 30–90%) that was assessed with invasive FFR between March and December 2018 at the Barts Heart Centre, London, United Kingdom, were enrolled. Patient/vessel exclusion criteria included left ventricular ejection fraction (LVEF) < 40% and invasive FFR performed in the culprit vessel of a patient presenting with acute coronary syndrome (ACS). All angiography cine images were acquired at 7.5fps and 7pps radiation-save mode. Angiography exclusion criteria comprised (1) aorto-ostial lesions (> 30% stenosis within ≤ 3 mm from the aorta); (2) previous coronary artery bypass grafts (3) coronary aneurysm of the assessed vessel; (4) bifurcation lesions with severe stenosis at the ostium of branch vessel (Medina 1,0,1 or 1,1,1); (5) inaccurate FFR measurement such as significant drift (> 0.02) and evidence of insufficient maximal hyperemia; (6) lack of 2 angiographic projections which are ≥ 30° for satisfactory QFR and vFFR analysis.

### Evaluation of angiography images for analysis

**Fig. 1:**
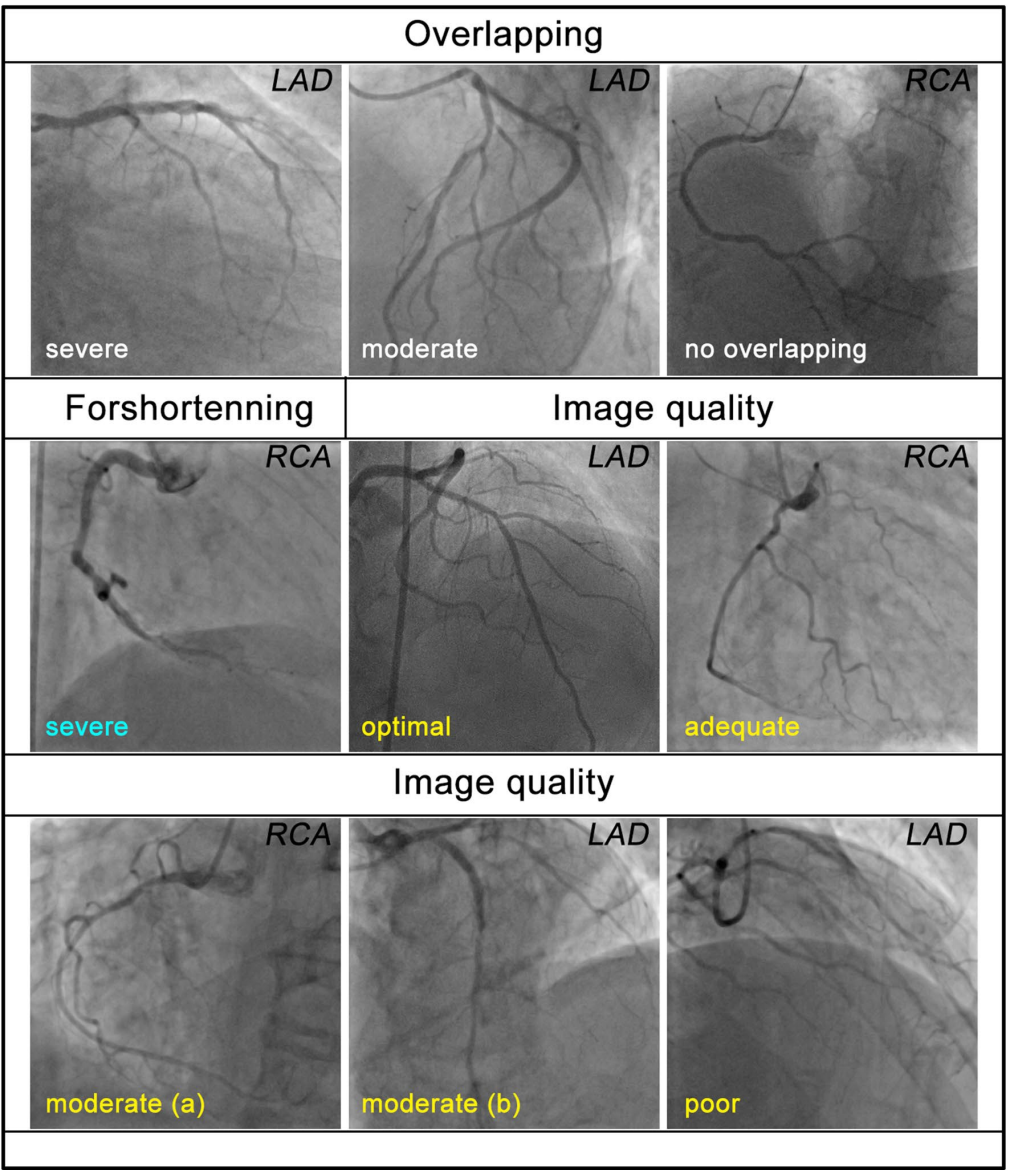
Typical sample images of different grades of each variable enrolled in the algorithmic excluding method (interrogated vessels are labeled at the right-up corner, image quality not acceptable is not shown due to lack of sample). Specific definition of each grade could be found in the supplements

All angiographic sequences included in the study were evaluated according to two different methods (described below) and excluded if their quality was deemed inadequate. For each vessel, two projections which are at least ≥ 30° apart were selected for QFR and vFFR analysis. Following that, two methods were used to examine the quality of angiography acquisitions. The first is the conventional manual (M group) method which relies on the operator’s expertise in analyzing the suitability of the images. Two experts (CJ and AR) independently assessed all the angiography images and excluded vessels with severe overlapping, tortuosity, foreshortening and poor vessel opacification. In the event of disagreement, a third expert’s (CB) opinion was sought for consensus. The same set of images was also analyzed using a new algorithm (A group) that included the following variables: vessel overlapping, foreshortening and image quality (Fig. 1). For each interrogated angiographic projection cine, these variables were classified into different severity grades with different weights. The weights of each interrogated angiographic cines were added, and the sums of the paired sets of projections were added to a final score (Table 1). The algorithm was first tested on a validation group (n = 51) to select a cut-off score beyond which the correlation of QFR/vFFR with invasive FFR could be improved. The algorithm was then applied to all the subjects with the selected cut-off, to exclude vessels with poor angiographic image quality.

**Table 1:** Weights of each factor enrolled for algorithmic image quality scoring

Factors and severity	Overlapping			Foreshortening		Image quality				
	Severe	Moderate	None	Severe	Others	Optimal	Adequate	Moderate	Poor	Not acceptable
Weights	3	1	0	3	0	-0.5	0	2	4	Exclude

### Coronary angiography and invasive pressure wire assessment

Coronary angiography was performed according to standard local practice. FFR measurement was taken during maximum hyperemia where the lowest value and drift during wire pullback were recorded. The aortic root pressure at the beginning of coronary angiography was recorded and used for vFFR analysis.

### QFR and vFFR analysis

QFR analysis was performed offline by using QAngio XA3D/QFR solution (Medis medical imaging system bv., Leiden, the Netherlands), while vFFR was calculated by CAAS workstation 8.0 (Pie Medical Imaging, Maastricht, the Netherlands) according to their well-establish protocols. To minimize the differences between the two systems, the same projection and end-diastolic frame (agreed by two experts) was selected and used for analysis in both. The distal reference point for vessel reconstruction during QFR/vFFR analysis was matched to the pressure wire sensor on coronary angiography while the proximal point was set at the visually normal proximal reference segment. For QFR, both fixed QFR (fQFR) and contrast QFR (cQFR) assessment were calculated. All the measurements were performed by 2 independent experts blinded to FFR results. vFFR analysis was performed at least 4 weeks following QFR analysis to minimize bias.

### Reproducibility

**Fig. 2:**
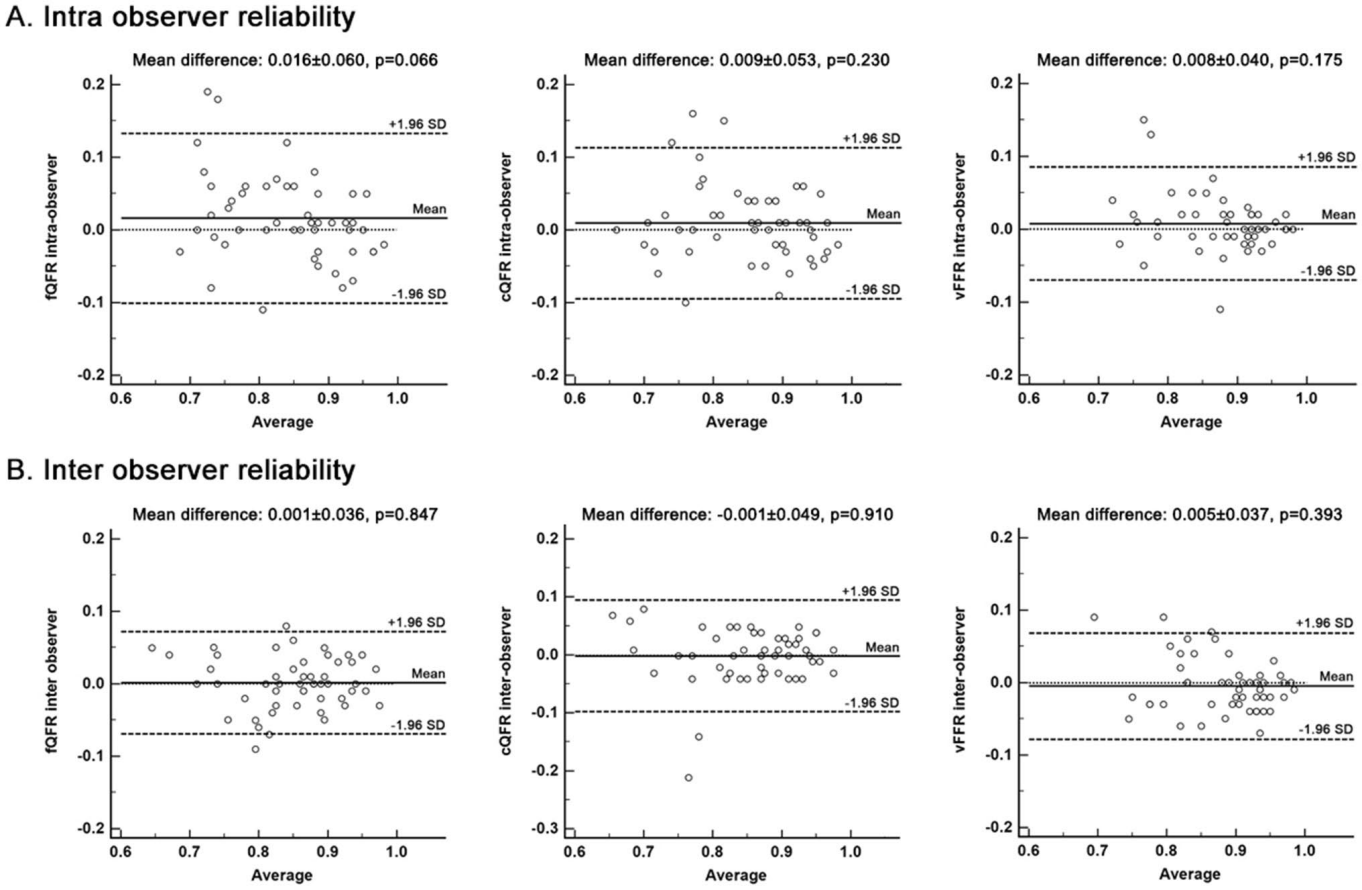
Bland–Altman plots of intra and inter observer reliability. For intra observer reliability, the mean difference between the 2 groups of measurements by observer 1 was 0.016 ± 0.060 (p = 0.066) for fQFR, 0.009 ± 0.053 (p = 0.230) for cQFR, and 0.008 ± 0.040 (p = 0.175) for vFFR, respectively. Inter-observer difference was also non-significant, with 0.001 ± 0.036 (p = 0.847) for fQFR, − 0.001 ± 0.049 (p = 0.910) for cQFR, and − 0.005 ± 0.037 (p = 0.393) for vFFR

The inter- and intra- observer reliability were tested to ensure reproducibility. 51 consecutive vessels were analyzed by 2 independent experts (CJ and AR), blinded to each other to test the inter-observer reliability. For the intra-observer reliability test, expert 1 (CJ) re-analyzed the same 51 vessels at 4 weeks after the first analysis. The intra- and inter-observer variability results are not statistically different as shown in Fig. 2.

### Statistical analysis

Continuous data, if normally distributed were expressed as mean ± SD, otherwise as median (interquartile range (IQR)). Categorical data were expressed as percentages. Data were analyzed on a per vessel bias for QFR or vFFR analysis. For mean comparison, a paired t-test was used. Bland–Altman plot analysis was used for evaluating the agreement between FFR and QFR/vFFR measurements, as well as for the intra- and inter-observer reliability. Pearson correlation was used to estimate the correlation between FFR and QFR/vFFR. Receiver operating characteristic (ROC) curve analysis was used to evaluate the diagnostic efficiency of QFR/vFFR with FFR ≤ 0.80 used as the gold standard to indicate functional significance. Sensitivity, specificity, accuracy, positive predicting value (PPV), negative predicting value (NPV), positive likelihood ratio ((+) LR) and negative likelihood ratio ((−) LR) were derived from ROC curve analysis. Paired ROC comparison was performed according to the DeLong method within the same group. For different exclusion method groups, a z-test was used for correlation and ROC curve comparison. All the analysis were performed using MedCalc Statistical Software version 18.2.1 (MedCalc Software bvba, Ostend, Belgium) and R software version 3.5.1 (R Foundation for Statistical Computing, Vienna, Austria). A p-value of < 0.05 was assumed to be statistically significant.

## Results

### Baseline clinical and lesion characteristics

A total of 134 vessels from 102 patients were screened, from which 22 vessels from 16 patients were excluded due to inaccurate FFR measurement or unsuitable coronary anatomy. Finally, 112 vessels from 86 patients were enrolled. The study baseline characteristics are summarized in Table 2. The mean age of the study population was 61.2 ± 14.3 years old, 31.4% were female, and 48.8% were diabetic.

**Table 2:** Baseline characteristics of study population (N = 86)

Age, years	61.2 ± 14.3	
Female	31.4	(27)
Hypertension	66.3	(57)
Diabetes	48.8	(42)
Hyperlipidemia	73.3	(63)
Current smoking	12.8	(11)
Previous MI	18.6	(16)
Previous PCI	34.9	(30)
Previous CABG	5.8	(5)
LVEF, %	54.8 ± 8.3	
Clinical presentation		
Stable angina or silent ischemia	79.1	(68)
Unstable angina	9.3	(8)
NSTEMI	10.5	(9)
STEMI	1.2	(1)
Interrogated vessels		
LM	1.2	(1)
LAD	72.1	(62)
Diagonal branch	1.2	(1)
LCX	10.5	(9)
Obtuse marginal branch	1.2	(1)
Ramus intermediate	1.2	(1)
RCA	12.8	(11)
QCA		
Lesion length, mm	18.4	[11.7–30.0]
Minimum lumen diameter, mm	1.4	[1.28–1.63]
Minimum lumen area, mm^2^	2.1	[1.6–2.6]
Diameter stenosis, %	44 ± 9	
Reference diameter, mm	2.6	[2.3–2.9]
Indices, mean ± SD		
FFR	0.85 ± 0.09	

Values are % (n), mean ± SD, n or median [interquartile range (IQR)]

*MI* myocardial infaction, *PCI* percutaneous coronary intervention, *CABG* coronary artery bypass surgery, *LVEF* left ventricular ejection fraction, *NSTEMI* non-ST-segment elevation myocardial infarction, *STEMI* ST-segment elevation myocardial infarction, *LM* left main, *LAD* left anterior descending, *LCX* left circumflex artery, *RCA* right coronary artery, *FFR* fractional flow reserve

### Angiography image quality evaluation

**Fig. 3:**
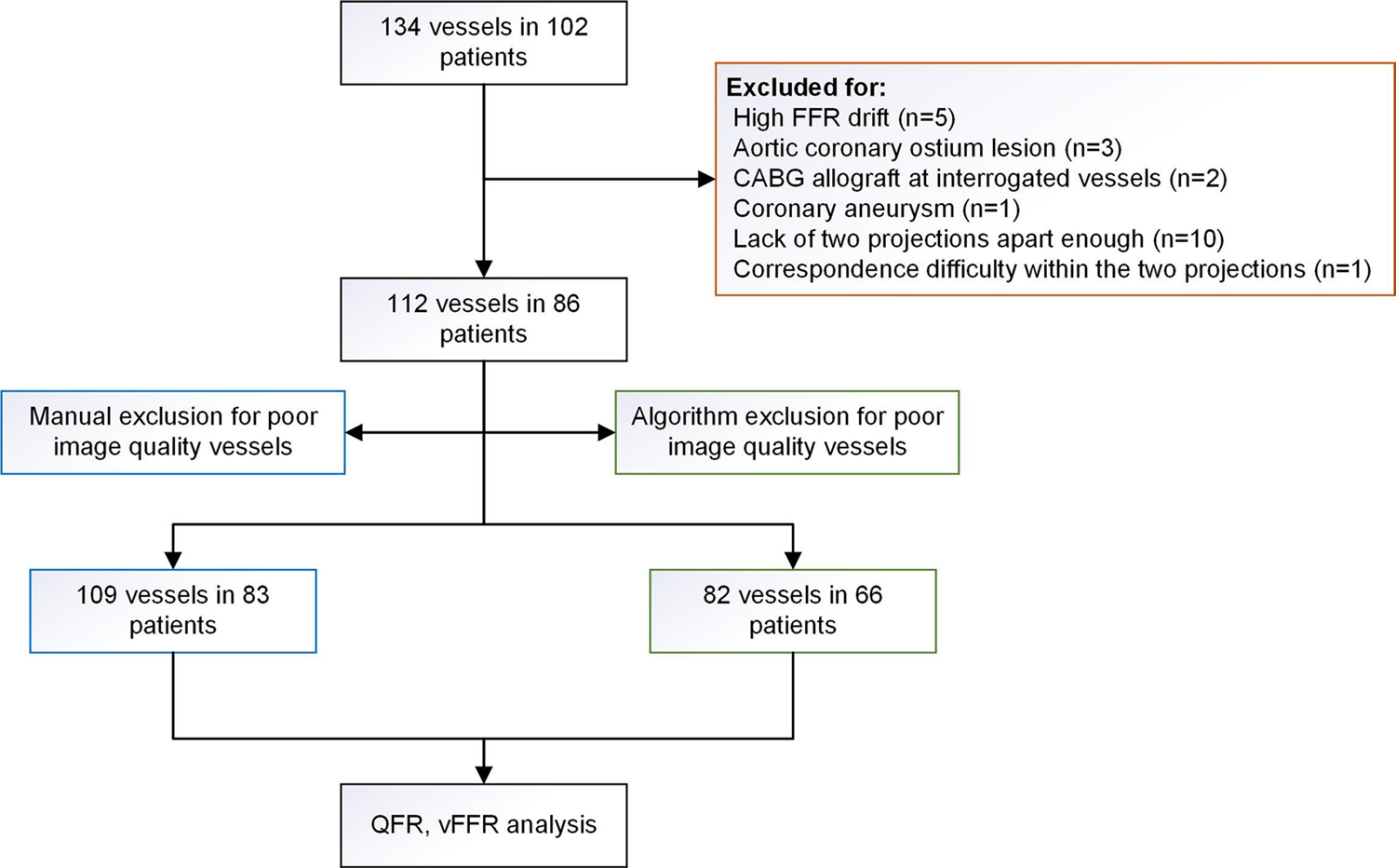
Study flowchart

In M group, 3 vessels were excluded due to severe overlapping (n = 1), poor filling of contrast (n = 1) and severe foreshortening (n = 1). In the validation cohort (n = 51) of A group, an increasing correlation between QFR/vFFR and FFR assessment was observed when excluding vessels using an algorithmic cut-off score of > 8, > 7, > 6, and > 5 points, respectively (Table 3). The algorithm was then applied to all the study subjects, the mean score of all the studied vessels was 4.32 ± 2.79, ranging from 0 to 9 points. The cut-off > 6 was chosen as a consideration of the best correlation with FFR for both QFR and vFFR in the validation cohort and maximum sample size saving. The A group finally enrolled 82 vessels from 82 patients. All the 3 vessels excluded in the M group were also excluded by the algorithmic method (Fig. 3).

**Table 3:** Correlation between QFR/vFFR and FFR by excluding cases with different cut-off score in validation cohort

Cut-off	N	Correlation coefficient with FFR		
		fQFR	cQFR	vFFR
(All cases)	51	0.6434	0.6029	0.3912^#^
> 8	47	0.6303	0.5795	0.4115^#^
> 7	42	0.7610	0.7192	0.5640^##^
> 6	38	0.7487	0.7107	0.6301
> 5	31	0.7846	0.7189	0.6149

^#^p < 0.01; ^##^p < 0.001; for all other correlation coefficient, p < 0.0001

### Agreement and correlation between QFR/vFFR and FFR

**Fig. 4:**
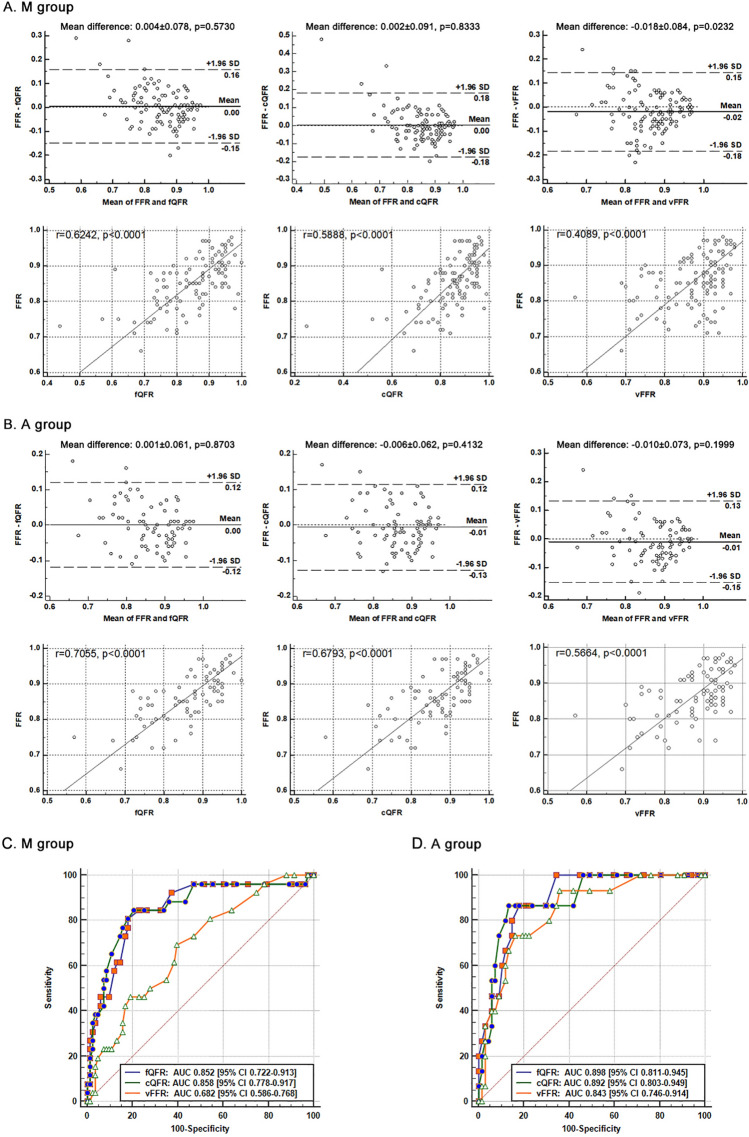
Agreement and correlation between QFR/vFFR and FFR, and ROC curves of identifying FFR ≤ 0.80. For both **a** and **b** panel, upper part: Bland–Altman plots of differences against the means; lower part: correlation between QFR/vFFR and FFR. For **c** and **d** panel, ROC curves of QFR/vFFR and their comparison within same group

There was no significant difference between the mean value of QFR and FFR measurements in both M and A group (mean difference with FFR: 0.004 ± 0.078, p = 0.5730 for fQFR, 0.002 ± 0.091, p = 0.8333 for cQFR in the M group, and 0.001 ± 0.061, p = 0.8703 for fQFR, − 0.006 ± 0.062, p = 0.4132 for cQFR in the A group). The mean value of vFFR measurements in the M group was significantly higher than FFR (mean difference: − 0.018 ± 0.084, p = 0.0232). However, in the A group, no statistically significant difference was observed between the two measurements (mean difference: − 0.010 ± 0.073, p = 0.1999). A good correlation was observed between QFR and FFR in both M and A group with the coefficient of 0.6242 (p < 0.0001) for fQFR, 0.5888 (p < 0.0001) for cQFR in the M group, and 0.7055 (p < 0.0001) for fQFR, 0.6793 (p < 0.0001) for cQFR in the A group. The correlation coefficient between vFFR and FFR was moderate in the M group (r = 0.4089, p < 0.0001), which was similar to cQFR (p = 0.0787) but significantly lower than fQFR (p = 0.0303), however, improved in the A group (r = 0.5664, p < 0.0001). Although the correlation coefficients between QFR/vFFR and FFR were higher in the A group than M group, there was no significant difference between the 2 exclusion methods (z = − 0.9844, − 1.0226, − 1.3989, p = 0.3249, 0.3065, 0.1619, for fQFR, cQFR and vFFR, respectively) (Fig. 4a, b).

### Diagnostic performance of QFR/vFFR on functional significant stenosis

FFR ≤ 0.80 was used as the gold standard to define functional significance. In the M group, both fQFR and cQFR showed good accuracy at detecting functional stenosis, while vFFR revealed moderate accuracy (AUC_fQFR_: 0.852 (95% CI 0.722–0.913), AUC_cQFR_: 0.858 (95% CI 0.778–0.917), AUC_vFFR_: 0.682 (95% CI 0.586–0.768)). AUC_vFFR_ was significantly lower than both cQFR and fQFR (AUC_fQFR_ vs AUC_vFFR_: z = 2.732, p = 0.0063; AUC_cQFR_ vs AUC_vFFR_: z = 2.782, p = 0.0054). In the A group, QFR showed high diagnostical performance (AUC_fQFR_: 0.898 (95% CI 0.811–0.945), AUC_cQFR_: 0.892 (95% CI 0.803–0.949)), while vFFR also showed good performance (AUC_vFFR_: 0.843 (95% CI 0.746–0.914)). No significant difference was observed within all the three assessments in this group according to the ROC curve comparisons (AUC_fQFR_ vs AUC_cQFR_: z = 0.322, p = 0.7478; AUC_fQFR_ vs AUC_vFFR_: z = 0.873, p = 0.3825; AUC_cQFR_ vs AUC_vFFR_: z = 0.741, p = 0.4585). vFFR had a better diagnostic performance in the A group than that in the M group (z = − 2.055, p = 0.0399), while QFR remained similar (fQFR: z = -0.794, p = 0.4271; cQFR: z = − 0.540, p = 0.5892) (Fig. 4c, d). Table 4 list the diagnostical performance of 3 different evaluation methods and the p values between the 2 different groups.

**Table 4:** Diagnostical performance of QFR/vFFR in different exclusion method groups

Groups	M group (N = 109)			A group (N = 82)			p value between M and A groups		
Measurement	fQFR	cQFR	vFFR	fQFR	cQFR	vFFR	fQFR	cQFR	vFFR
AUC	0.852	0.858	0.682^*^	0.898	0.892	0.843^#^	0.4271	0.5892	**0.0399**
	(0.722–0.913)	(0.778–0.917)	(0.586–0.768)	(0.811–0.945)	(0.803–0.949)	(0.746–0.914)			
Accuracy (%)	80.73	83.49	72.48	82.93	87.80	82.93	0.6980	0.4058	0.0904
	(72.07–87.66)	(75.16–89.91)	(63.10–80.60)	(73.02–90.34)	(78.71–93.99)	(73.02–90.34)			
Sensitivity (%)	73.08	65.38	34.62	73.33	73.33	60.00^##^	0.9693	0.2417	**0.0005**
	(52.21–88.43)	(44.33–82.79)	(17.21–55.67)	(44.90–92.21)	(44.90–92.21)	(32.29–83.66)			
Specificity (%)	83.13	89.16	84.34	85.07	91.04	88.06	0.7183	0.6694	0.4653
	(73.32–90.46)	(80.41–94.92)	(74.71–91.39)	(74.26–92.60)	(81.52–96.64)	(77.82–94.70)			
PPV (%)	57.58	65.38	40.91	52.38	64.71	52.94	0.4757	0.9236	0.0996
	(44.37–69.78)	(48.97–78.81)	(25.08–58.88)	(36.52–67.77)	(44.62–80.66)	(34.24–70.85)			
NPV (%)	90.79	89.16	80.46	93.44	93.85	90.77^#^	0.5069	0.2593	**0.0495**
	(83.86–94.93)	(82.82–93.34)	(75.41–84.68)	(85.96–97.07)	(86.78–97.25)	(84.02–94.84)			
(+)LR	4.33	6.03	2.21	4.91	8.19^##^	5.02^##^	0.1743	**0.0001**	** < 0.0001**
	(2.55–7.37)	(3.06–11.87)	(1.07–4.57)	(2.57–9.39)	(3.60–18.63)	(2.33–10.86)			
(−)LR	0.32	0.39	0.78	0.31	0.29	0.45	0.9847	0.8513	0.2895
	(0.17–0.61)	(0.23–0.66)	(0.58–1.04)	(0.13–0.73)	(0.13–0.68)	(0.24–0.85)			

Bold represent the statistically significant p values

*p < 0.01, compared to fQFR/cQFR in the same group. ^#^p < 0.05, compared to same method in the M group. ^##^p < 0.001, compared to same method in the M group

## Discussion

Our study confirmed the feasibility of two commercially available angiography derived FFR solutions (QFR and vFFR) for assessing the functional significance of intermediate coronary stenosis using angiographic images performed under radiation save mode (7.5 fps and 7pps). Under such conditions, QFR showed good agreement and correlation with the invasive pressure wire derived FFR by both conventional manual exclusion and our newly developed algorithm of excluding poor quality images. The results from our study are in line and confirm the good diagnostical performance of previous QFR studies with high sensitivity, specificity and accuracy when compared with invasive FFR [6, 11, 12]. The manual exclusion method which relies on the expert’s visual interpretation of suitability of angiographic images revealed that vFFR showed a moderate correlation with FFR and diagnostical performance of detecting functional stenosis. However, this significantly improved when the new algorithm was used, with which the diagnostic accuracy, agreement and correlation of vFFR was similar to QFR when compared to invasive FFR.

Radiation exposure during coronary angiography and PCI is a major concern for patients and interventional cardiologists. The main principle of radiation exposure should be “as low as reasonably achievable”. This is to reduce the effects of radiation on superficial skin reactions and cancer risks to patients and catheterization suite staffs [16]. Modern X-ray scanners deliver X-ray energy in pulses, therefore reducing the pulse rate from the conventional 15 pps to a lower value is a good way of reducing radiation exposure [13]. Another method of reducing radiation exposure is to reduce the conventional 15fps frame rate that is routinely used during fluoroscopic and cine-acquisition [14]. Although reduction of pulse rates from 15 to 10pps has little impact on image quality [13], extremely lower pps (< 10pps) could result in a decrease in perceived resolution and contrast [16]. Also, the reduction of frame rates from 15fps to 7.5fps has been identified to reduce the image quality [14]. A combination of a reduction in both fps and pps is commonly used for reducing radiation, however, with a cost of reducing angiographic image quality, at both contrast and resolution level [17].

All the angiography derived FFR assessments require 3D-QCA reconstruction derived from at least 2 angiographic projections. The current recommendation for both QFR and vFFR is to use angiographic images acquired with 15 fps, without specific clarification on pulse rate setting. This is to ensure good accuracy of vessel contour detection, which provides the required geometry of a vessel for 3-dimensional reconstruction. Therefore, this would limit the application to prospectively acquired angiograms and restrict the use in a retrospective analysis of angiograms performed with contemporary radiation protection parameters. To our knowledge, this is the first study that has examined and confirmed the efficacy of angiography derived FFR software using angiography images acquired under 7.5fps and 7pps radiation save mode.

So far, all the validation studies of angiography derived FFR against invasive FFR have focused on the manual exclusion of poor-quality angiograms prior to analysis. The exclusion rates varied, from 0.3 to 8% for QFR [6, 11, 12, 18] and up to 13% of the screened cases for vFFR [7], depending on the study design and the requirements of angiography image acquisition. Often, the excluded cases are labelled as ‘poor image quality’ rather than a specific exclusion reasoning or definition, not only in validation studies of QFR/vFFR, but also other angiography derived FFR validation trials [10, 19]. In our current study, the exclusion rate in the M group should theoretically be higher than those studies using normal frame and pulse rate settings because of the relatively lower image quality, but in fact, was actually similar (2.7%). This may because that the observers have been used to these low dose images’ characteristics and therefore subjectively think that they’re suitable for analysis, which indicates the manual exclusion method may not be very reliable. Thus, a more objective and reliable exclusion method needs to be considered.

Our new algorithmic exclusion method, for the first time, provides a new quantitative method of screening angiographic images that are suitable for angiography derived FFR measurement. By using a cut-off score of 6 points, this algorithm helps to improve the performance of vFFR, both in agreement with FFR and in detecting FFR ≤ 0.80. The algorithm also had slight improvement when applied to the QFR analysis but without statistical significance. Although the exclusion rate is relatively high, our algorithm provided a well-defined methodology to select angiograms with adequate quality for angiography derived FFR assessment, especially when the angiograms have been acquired under radiation save mode and when vFFR was used for analysis. Cases excluded by the algorithm should be considered to use invasive FFR to detect functional stenosis for accuracy, and avoiding assessment with vFFR.

Currently, no head-to-head comparisons have been performed between angiography derived FFR software packages. Although the correlation coefficient and AUC value in the FAST study of vFFR was higher than those in FAVOR series studies of QFR, a recent meta-analysis showed that the computational approaches and mathematical formula based techniques did not influence the diagnostic accuracy of angiography derived FFR [5]. Thus, the performance of vFFR could theoretically be similar to QFR. The application of our algorithm to select angiograms with adequate quality is useful where vFFR showed similar performance at detecting functional ischemia with QFR. However, vFFR was inferior to QFR when the angiographic images were manually selected.

**Fig. 5:**
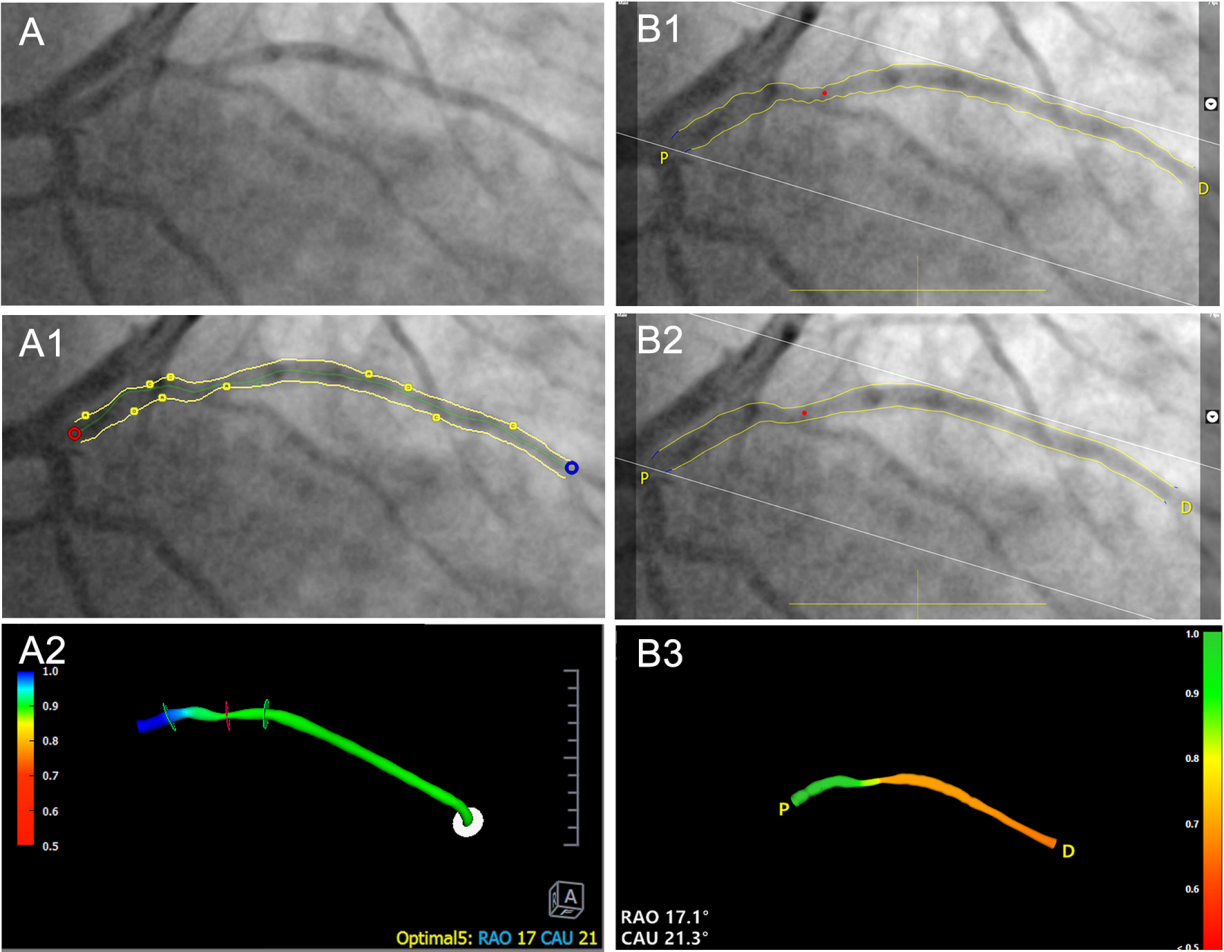
Vessel contour detection with necessary manual modifications. Same vessel (an intermediate ramus) was assessed by both QFR and vFFR software package. **A** Initial frame without any projections. **A1** Semi-automatic detection of vessel contours with necessary manual modifications by QFR software package. **A2** 3D reconstruction of the vessel by QFR software package. **B1** semi-automatic detection of vessel contours with necessary manual modifications by vFFR software package, the contours are not so smooth and jagged. **B2** Same vessel contours after being modified with “Hard correction” tool provided by the software package, the vessel contours become smoother. **B3** 3D reconstruction of the vessel by vFFR software package, the reconstructed vessel wall is not as smooth as that in QFR software

One of the reasons of the poorer performance of vFFR in manual exclusion group may be due to its vessel contour detecting algorithm. Figure 5 shows a vessel analyzed in both software packages. Vessel contours were semi-automatically detected with minimal modifications. The QFR contour detection algorithm appears to be much smoother than vFFR. The automated detection can be modified manually in vFFR but this can lead to errors and higher observer variability. Table 5 lists the 3D-QCA results provided by both QFR and vFFR software package using their default lesion detections on the interrogated vessels. The minimal lumen diameter, minimal lumen area and reference diameters generated by QFR software package in the M group were all significantly lower than those by vFFR software package, while the diameter stenosis percentage was significantly higher. In the A group, the minimal lumen diameter, reference diameters generated by QFR were still lower than those by vFFR, the diameter stenosis percentage remained higher, however, there was no significant difference at minimal lumen area generated by the two different packages. This may indicate the reason of the statistically higher vFFR assessment in the M group while QFR revealed similar with FFR, as vFFR provided bigger mean minimal lumen area, while in the A group, similar minimal lumen area was provided by both QFR and vFFR packages.

**Table 5:** The 3D reconstruction projections of both QFR and vFFR in M and A group

		MLD	MLA	RD	DS%
M group	QFR package	1.45 ± 0.33	2.23 ± 1.09	2.60 ± 0.48	44.1 ± 8.8
	vFFR package	1.78 ± 0.39	2.59 ± 1.17	3.00 ± 0.78	37.7 ± 16.6
	p value	< 0.0001	0.0248	< 0.0001	0.0006
A group	QFR package	1.45 ± 0.35	2.24 ± 1.17	2.59 ± 0.49	44.2 ± 9.2
	vFFR package	1.77 ± 0.40	2.59 ± 1.19	2.96 ± 0.76	37.1 ± 17.2
	p value	< 0.0001	0.0663	0.0001	0.0015

Data were provided by both QFR and vFFR software package using their default lesion detections on the interrogated vessels

*MLD* minimal lumen diameter, *MLA* minimal lumen area, *RD* reference diameter, *DS%* diameter stenosis percentage

Apart from the potential poorer image quality, angiographic images acquired with low frame rate influences the frame counting during QFR analysis. Frame counting is essential for cQFR which has shown to be superior to fQFR or adenosine-flow QFR (aQFR) [6]. In our current study, cQFR revealed no additional advantage compared with fQFR. In the cQFR processing, the shortest thrombolysis in myocardial infarction (TIMI) frame count is essential for calculating the contrast medium transport time in the interrogated vessel, which is one of the key factors to calculate hyperemic flow velocity (HFV) [6, 20]. Hence, lower frame rate results in a more extensive time interval, which decrease the precision of contrast medium transport time, therefore influences the precision of HFV, and in turn the accuracy of cQFR. The lowest frame rate used in QFR validation studies was 12.5fps and is currently the recommended minimum by the software [12]. Unlike cQFR, fQFR uses a fixed empiric HFV of 0.35 m/s, therefore lower frame rate does not affect the calculation of fQFR. Our current study revealed that both fQFR and cQFR had a similar performance at detecting functional stenoses, this may allow time to be saved by focusing on fQFR when low frame rate radiation save mode angiograms are used for analysis.

## Limitations

This study is a retrospective study, 22 cases were excluded due to lack of 2 suitable projections for QFR/vFFR analysis and inaccurate FFR measurement, this may cause bias. A prospective multi-center study with a larger patient cohort is needed to validate our algorithm and assess the effects of angiography derived FFR. Also, this study only evaluated the performance of QFR/vFFR under 7.5fps/7pps angiograms, other radiation saving mode (e.g. 7.5fps/15pps or 15fps/7pps, etc.) angiograms should be further analyzed to draw a clear picture of the performance of QFR/vFFR under such conditions.

## Conclusion

Angiography derived FFR assessment of intermediate coronary stenosis is feasible under radiation save mode—7.5fps and 7pps coronary angiography. In these cases, the newly developed algorithm helps to exclude vessels that may not be suitable for angiography derived FFR analysis, thus improving the diagnostic accuracy of these commercially available software packages. With 7.5fps angiography, cQFR reveals no additional advantage compared to fQFR, so fQFR could be the first choice for evaluating such cases.

## Supplementary Information

Below is the link to the electronic supplementary material.

Supplementary file1 (DOCX 23 KB)
