# Supplementary material for: Diagnostic accuracy of quantitative flow ratio (QFR) and vessel fractional flow reserve (vFFR) estimated retrospectively by conventional radiation saving X-ray angiography
Source: Int J Cardiovasc Imaging. 2021 Jan 16;37(5):1491–501. doi: 10.1007/s10554-020-02133-8 (PMC8105229; doi:10.1007/s10554-020-02133-8)
Supplement: Supplementary file 1 — Supplementary file1 (DOCX 23 KB) [file 10554_2020_2133_MOESM1_ESM.docx]

**Supplements**

1. **Algorithmic scoring and excluding method:**
2. **Impact factors and grade (Figure 1):**

For each interrogated angiographic projection cine, the factors below were considered as the main impact factors and classified into different severity grades according to previous studies^[1-3]^ and our experience. Unlike other studies, we considered tortuosity not to affect the efficacy of QCA reconstruction if not accompany with overlapping or foreshortening and didn’t take this factor in.

1. **Overlapping:** Overlapping of other vessel(s) on the interrogated vessel will cause difficulty on vessel contour identifying, which is critically important for angiography derived FFR assessment. Hence, we made a classification of overlapping severity.

**Severe:** Overlapping within the lesion segment for >1/3 lesion length during the diastole, or overlapping at the reference segment with: a. a length >5mm or b. a length >3mm but lack of an additional reference segment can be used for analysis.

**Moderate:** Overlapping presented in the lesion segment, but is ≤1/3 of lesion length during the diastole, or overlapping at the interrogated vessel including the reference segment (≤5mm in length, or ≤3mm in length when there’s no additional reference segment can be used for analysis), or a combination of both two criteria listed above.

**No overlapping:** No overlapping during the diastole.

1. **Foreshortening:** Foreshortening can mask the extend and severity of a lesion, here we classify severe “foreshortening”, if the lumen borders of either lesion or reference segment cannot be accurately annotated.
2. **Image quality**: The image quality could influence lumen annotation and affect angiography derived FFR assessment. We used a modified classification^[4]^ to grade the image quality using 3 parameters including vessel opacification, vessel contour clarity and contrast to background ratio:

**Optimal quality:** complete opacification of the entire length of the interrogated vessel throughout diastole, the vessel contours are sharp with adequate contrast to the background ratio

**Adequate quality:** complete or almost complete opacification of the vessel throughout diastole, but the vessel contours are slightly vague with good contrast to background ratio.

**Moderate quality:** a. complete or almost complete opacification of the vessel throughout diastole, but the vessel contours are very vague; b. moderate opacification of the vessel throughout diastole, the vessel contours are slightly vague.

**Poor quality:** moderate opacification of the vessel throughout diastole with very vague vessel contours.

**Not acceptable (exclusion):** absence of contrast opacification

1. **S****cores and the algorithmic system:**

Different weights were given to every severity grade of each variable initially according to the researcher’s experience. For scoring, the defined weights of each projection after the judgement was added, and the sum of 2 combined projections was considered as the total score of the interrogated vessel. The weights were adjusted several times until a best correlation between the total score and absolute difference between fQFR/cQFR/vFFR and FFR was identified (r_ABS(FFR-fQFR)_=0.3761, p=0.0065, r_ABS(FFR-cQFR)_=0.4118, p=0.0027, r_ABS(FFR-vFFR)_=0.2220, p=0.1174). **Table 1** represents the final weights selected in the current study.

1. **Validation of algorithmic excluding method**

A validation cohort (n=51) was used for evaluating the algorithmic excluding method of poor angiographic images. The total score of this cohort varied from 0-9 points. **Table 3** presents the correlation between QFR/vFFR and FFR while using different cut-off scores to exclude vessels with poor image quality. The peak of the correlation coefficient started at cut-off >7 for QFR and >6 for vFFR.

**References**

[1] Tu S, Westra J, Yang J, et al. Diagnostic Accuracy of Fast Computational Approaches to Derive Fractional Flow Reserve From Diagnostic Coronary Angiography: The International Multicenter FAVOR Pilot Study. JACC Cardiovasc Interv. 2016. 9(19): 2024-2035.

[2] Masdjedi K, van Zandvoort L, Balbi MM, et al. Validation of 3-Dimensional Quantitative Coronary Angiography based software to calculate Fractional Flow Reserve: Fast Assessment of STenosis severity (FAST)-study. EuroIntervention. 2019 .

[3] Westra J, Andersen BK, Campo G, et al. Diagnostic Performance of In-Procedure Angiography-Derived Quantitative Flow Reserve Compared to Pressure-Derived Fractional Flow Reserve: The FAVOR II Europe-Japan Study. J Am Heart Assoc. 2018. 7(14).

[4] Chow W, Bing R, Kanawati J, et al. A Comparison of Image Quality Using Radial vs Femoral Approaches in Patients Undergoing Diagnostic Coronary Angiography. J Invasive Cardiol. 2018. 30(11): 411-415.
